# Bovine Coronavirus Co-infection and Molecular Characterization in Dairy Calves With or Without Clinical Respiratory Disease

**DOI:** 10.3389/fvets.2022.895492

**Published:** 2022-05-25

**Authors:** Ana Paula S. Frucchi, Alais M. Dall Agnol, Dalton E. Bronkhorst, Edsel A. Beuttemmuller, Amauri A. Alfieri, Alice F. Alfieri

**Affiliations:** ^1^Laboratory of Animal Virology, Department of Veterinary Preventive Medicine, Universidade Estadual de Londrina, Londrina, Brazil; ^2^Multi-User Animal Health Laboratory, Molecular Biology Unit, Department of Veterinary Preventive Medicine, Universidade Estadual de Londrina, Londrina, Brazil; ^3^National Institute of Science and Technology for Dairy Production Chain (INCT–LEITE), Universidade Estadual de Londrina, Londrina, Brazil

**Keywords:** calf, BRD, spike protein, *Pasteurella multocida*, *Mannheimia haemolytica*

## Abstract

Bovine respiratory disease (BRD) is considered a major cause of morbidity and mortality in young calves and is caused by a range of infectious agents, including viruses and bacteria. This study aimed to determine the frequency of viral and bacterial pathogens detected in calves with BRD from high-production dairy cattle herds and to perform the molecular characterization of N and S1 genes in identified bovine coronavirus (BCoV) strains. Nasal swabs were collected from 166 heifer calves, namely, 85 symptomatic and 81 asymptomatic calves aged between 5 and 90 days, from 10 dairy cattle herds. Nasal swabs were evaluated using molecular techniques for the identification of viruses (BCoV, bovine alphaherpesvirus 1, bovine viral diarrhea virus, bovine parainfluenza virus 3, and bovine respiratory syncytial virus) and bacteria (*Pasteurella multocida, Mannheimia haemolytica, Histophilus somni*, and *Mycoplasma bovis*). In addition, five and two BCoV-positive samples were submitted to N and S1 gene amplification and nucleotide sequencing, respectively. The frequency of diagnosis of BCoV was higher (56%, 93/166) than the frequency of *P. multocida* (39.8%, 66/166) and *M. haemolytica* (33.1%, 55/166). The three microorganisms were identified in the calves of symptomatic and asymptomatic heifer calve groups. All other pathogens included in the analyses were negative. In the phylogenetic analysis of the S1 gene, the Brazilian strains formed a new branch, suggesting a new genotype, called # 15; from the N gene, the strains identified here belonged to cluster II. This study describes high rates of BCoV, *P. multocida*, and *M. haemolytica* in heifer calves from high-production dairy cattle herds with BRD. Additionally, the molecular characterization provides evidence that the circulating BCoV strains are ancestrally different from the prototype vaccine strains and even different BCoV strains previously described in Brazil.

## Introduction

Respiratory disorders are considered a major cause of morbidity and mortality in young calves worldwide (1–3). Bovine respiratory disease (BRD) is a multifactorial disorder caused by a range of infectious agents that affect the respiratory system (4). The BRD has a direct impact on welfare and animal production rates due to the lower growth, occurrence of secondary diseases, and costs associated with of prophylaxis, treatment, and death of the animals (2, 5).

BRD can be caused by a single infection or mixed infections as a result of the interaction of several viral and bacterial pathogens. Among the most common viral pathogens are bovine coronavirus (BCoV), bovine viral diarrhea virus (BVDV), bovine alphaherpesvirus 1 (BoAHV1), bovine respiratory syncytial virus (BRSV), and bovine parainfluenza virus 3 (BPIV-3) (1, 6, 7). Viral pathogens are mainly present in primary respiratory tract infections. The bacterial agents frequently found are *Mannheimia haemolytica, Pasteurella multocida, Histophilus somni*, and *Mycoplasma bovis* (8–10) which can act as primary or more common as secondary pathogens.

Among the infectious agents, BCoV is one of the main viruses associated with BRD and diarrhea in calves of dairy and beef cattle herds worldwide (11–14). Coronaviruses are enveloped, pleomorphic, and have a single-stranded, positive-sense, RNA virus, with a genome size of approximately 27.6-32 kb. BCoV belongs to the order *Nidovirales, Coronaviridae* family, *Betacoronavirus* genus, and *Betacoronavirus 1* species (15).

Viral particles are formed by five structural proteins: spike glycoprotein (S), hemagglutinin-esterase glycoprotein (HE), envelope protein (E), membrane protein (M), and nucleocapsid protein (N) (12). The N protein is internal to the viral envelope and is associated with viral RNA. Glycoprotein S projects out of the viral envelope and is subdivided into S1 and S2. The S1 subunit is responsible for virus binding to host cell receptors and contains the dominant neutralizing epitopes; thus, it is a target of molecular epidemiology studies (16). The S2 subunit mediates viral membrane fusion (12).

The association of BCoV with enteric disorders is frequently reported in cattle herds from Brazil, causing important productive and economic problems (17–20). However, few studies conducted in Brazil report the association of BCoV infection with BRD in calves (6, 21, 22). This study aimed to determine the frequency of viral and bacterial pathogens detected in calves with respiratory disease from high-production dairy cattle herds and perform the molecular characterization of N and S1 genes in BCoV strains identified.

## Materials and Methods

### Dairy Cattle Herds and Animals Sampled

Ten dairy cattle herds located in the central-eastern region, in the state of Paraná, southern Brazil were evaluated. This region has a humid temperate climate with temperate summers. All dairy cattle herds had a high technological level and significant milk production with average production from 37 to 42 L/milk/day/cow. Herds were composed of animals of Holstein and Jersey breeds or crossbreeds of both. A compost barn system was used by all farms. None of the herds performed vaccination of calves to control BRD and neonatal diarrhea. On all farms, the calves were kept in collective pens and were fed in a single automatic feeding system with milk from the herd or milk replacer.

In all 10 evaluated herds, heifer calves with clinical signs of BRD (symptomatic) as cough, nasal or ocular discharge, pale mucous membranes, and apparent breathing difficulties, and heifer calves without clinical signs (asymptomatic) were identified. After the identification of calves, nasal swabs were collected from symptomatic heifer calves, and so as a control group, were also collected from asymptomatic heifers calves in each herd evaluated. Nasal swab samples were collected from 166 heifer calves aged 5 to 90 days, including 85 symptomatic (presence clinical signs of BRD calves) and 81 asymptomatic calves (control calves—absence the clinical signs of BRD) calves. In this cross-sectional study, only a single collection of nasal swabs was performed per herd being in six herds in 2018, two herds in 2019, and two herds in 2020. The sampling of 10 herds was always carried out in the winter months (June to August) in the Southern Hemisphere. Only heifer calves that had not received any therapy for BRD treatment were selected for nasal swab collection. On all farms evaluated, symptomatic calves were kept together with asymptomatic calves. The swab nasal samples were placed in sterile tubes, transported in ice baths to the laboratory, and stored at−80 °C until processing.

### Nucleic Acid Extraction and Molecular Detection of Infectious Pathogens

Nucleic acid was extracted from 500 μL aliquots of nasal swab samples with 0.01 M phosphate-buffered saline, pretreated with sodium dodecyl sulfate and proteinase K and incubated at 56 °C for 30 min at final concentrations of 1% (v/v) and 0.2 mg/mL, respectively. The samples were then processed using the silica/guanidine isothiocyanate method (23). The extracted nucleic acid was eluted in 50 μL of ultrapure diethylpyrocarbonate (DEPC)-treated water (Invitrogen™ Life Technologies, Carlsbad, CA, USA) and stored at−80 °C until use for molecular analysis.

The presence of infectious agents associated with BRD was evaluated using molecular diagnostic assays. The target genomic region, amplification technique, and amplified product size are described in Table 1. In addition, for the molecular classification of BCoV, two samples positive for BCoV in the N gene were selected and submitted to a new RT-PCR with specific primers for the S1 gene. Aliquots of sterile ultrapure water were included as negative controls in all procedures. Prototype strains such as Los Angeles, NADL, A51908, SF4/32, and Mebus adapted in cell culture (MDBK) were included in the reactions as positive controls for BoHV-1, BVDV, BRSV, BPIV-3, and BCoV, respectively. Biological samples previously known as positive were included as positive controls for *H. somni, P. multocida, M. bovis* (6), and *M. haemolytica* (22).

**Table 1:** Target genomic region, amplification techniques and amplified product size to detect respiratory infectious agents in nasal swab samples of dairy heifer calves.

**Infectious agent**	**Target genomic region**	**Amplification technique**	**Amplified product size (bp)**	**Reference**
BCoV	N	RT-semi-nested-PCR	251	(20)
	S1	RT-PCR	2,719	(19)
BVDV	5′ UTR	RT-PCR	288	(24)
BRSV	G	RT-nested-PCR	371	(25)
BPIV-3	HN	RT-PCR	647	(26)
BoAHV1	C	PCR	425	(27)
*M. haemolytica*	lktA-artJ	PCR	385	(8)
*H. somni*	16S	PCR	408	(28)
*P. multocida*	KMT1	PCR	460	(29)
*M. bovis*	16S−23S	nested-PCR	488	(30)

*BCoV, bovine coronavirus; BVDV, bovine viral diarrhea virus; BRSV, bovine respiratory syncytial virus; BPIV-3, bovine parainfluenza virus 3; BoAHV1, bovine alphaherpesvirus 1; M. haemolytica, Mannheimia haemolytica; H. somni, Histophilus somni; P. multocida, Pasteurella multocida; M. bovis, Mycoplasma bovis*.

### Sequence Analysis

To confirm the specificity of the PCR amplicons different positive samples were randomly selected for nucleotide (nt) sequencing analyses. The amplicons were purified using a PureLink® Quick Gel Extraction and PCR Purification Combo Kit (Invitrogen® Life Technologies, Carlsbad, CA, USA) and quantified using Qubit® Fluorometer (Invitrogen® Life Technologies, Eugene, OR, USA). Direct sequencing was performed using a BigDye® Terminator v3.1 Cycle Sequencing Kit (Applied Biosystems®, Foster City, CA, USA) with the forward and reverse primers in a 3500 Genetic Analyzer sequencer. Sequence quality analyses and consensus sequences were obtained using Phred and CAP3 software (http://asparagin.cenargen.embrapa.br/phph/), respectively. Similarity searches were performed with nt sequences deposited in the GenBank database using the Basic Local Alignment Search Tool software (https://blast.ncbi.nlm.nih.gov/Blast.cgi). Multiple and pairwise alignments with strains available in GenBank were performed with MEGA software version 7.0.26 (31), and the nt and amino acid (aa) sequence identity matrices were constructed using BioEdit software version 7.2.5 (32). Phylogenetic trees based on nt sequences of the N and S1 genes of BCoV were obtained using the maximum likelihood method with the general time reversible model (33) using MEGA software version 7.0.26 (31). The bootstrapping probabilities were calculated using 1,000 replicates.

## Results

### Detection of Infectious Pathogens

Only BCoV, *P. multocida*, and *M. haemolytica* were identified in nasal swabs of symptomatic and asymptomatic heifer calves from dairy cattle herds evaluated. All other viruses and bacteria that were tested (BoAHV1, BVDV, BPIV3, BRSV, *M. bovis*, and *H. somni*) were negative.

The BCoV identification frequency was higher (56%, 93/166) than the frequencies of *P. multocida* (39.8%, 66/166) and *M. haemolytica* (33.1%, 55/166) (Table 2). The three microorganisms were identified in nasal swabs from symptomatic (BCoV: 54.1%; 46/85; *P. multocida*: 41.2%; 35/85; and *M. haemolytica*: 28.2%; 24/85) and asymptomatic (BCoV: 58%; 47/81, *P. multocida*: 38.3%; 31/81, and *M. haemolytica*: 38.3%; 31/81) calves. In 55/166 (33.1%) nasal swab samples, no etiological agent was detected, of which 26/55 (47.3%) were from symptomatic and 29/55 (52.7%) were from asymptomatic calves. The distribution of infectious agents identified in heifer calves with (symptomatic) and without (controls) clinical respiratory signs by herd is presented in Supplementary Table S1.

**Table 2:** Infectious agents identified in the upper respiratory tract of heifer calves, with and without (controls) clinical respiratory signs, from high production dairy cattle herds.

**Infectious agents**	**Herds (*****n*** **=** **10)**		**Positive calves (%)**		
	**Positive**	**Negative**	**BRD** **(*n* = 85)**	**Control** **(*n* = 81)**	**Total** **(*n* = 166)**
BCoV	9	1	46 (54.1)	47 (58.0)	93 (56.0)
*P. multocida*	9	1	35 (41.2)	31 (38.3)	66 (39.8)
*M. haemolytica*	3	7	24 (28.2)	31 (38.3)	55 (33.1)

*BRD, Bovine respiratory disease; BCoV, bovine coronavirus; P. Multocida, Pasteurella multocida; M. haemolytica, Mannheimia haemolytica*.

In terms of to the dairy cattle herds evaluated, BCoV and *P. multocida* were identified in 9/10 (90%), while *M. haemolytica* was detected in 3/10 (30%). One of the herds was negative for BCoV and *P. multocida*; however, it was positive for *M. haemolytica*. Interestingly 52/55 (94.5%) positive samples for *M. haemolytica* belonged to only two herds that had a total infection rate of 77.1 and 64.1% (27/35 and 25/39, respectively), including the calves with BRD and controls.

Regarding the profile of etiological agents found in nasal swabs from positive calves, the association of viral and bacterial infections was more frequent (59/111; 53.1%), followed by only viral infection (34/111; 30.6%) and by only bacterial infection (18/111; 16.2%) (Table 3). Mixed infection was identified in 9/10 (90%) dairy cattle herds evaluated. Regarding mixed infections per herd, 8/10 (80%) had viral and bacterial infections, while 1/10 (10%) had infection only by virus and another (10%) had infection only by bacteria (Table 2).

**Table 3:** Distribution of the infectious agents identified in the upper respiratory tract of heifer calves of dairy cattle herds with bovine respiratory disease according to the type of infection.

**Infection type**	**Infectious agents**	**N** **°** **positive calves**		
		**Symptomatic**	**Asymptomatic**	**Total**
Single	BCoV	19	15	34
	*P. multocida*	5	2	7
	*M. haemolytica*	3	1	4
Double	BCoV + *P. multocida*	10	5	15
	BCoV + *M. haemolytica*	2	5	7
	*P. multocida* + *M. haemolytica*	4	3	7
Triple	BCoV + *P. multocida* + *M. haemolytica*	16	21	37
**Total of positive calves**		**59**	**52**	**111**

*BCoV, bovine coronavirus; P. Multocida, Pasteurella multocida; M. haemolytica, Mannheimia haemolytica*.

### Sequence Analysis

The nt sequence analyses confirmed the specificity of BCoV, *P. multocida*, and *M. haemolytica* amplicons obtained from the different nasal swab samples.

Five BCoV strains identified from three different herds were selected for molecular analysis of the N protein gene. Of these, two strains (BRA/PR-227-840/2018-N and BRA/PR-227-843/2018-N) came from a herd sampled in 2018, two strains (BRA/PR-323-425/2019-N and BRA/PR-323-1543/2019-N) came from a herd sampled in 2019, and, one strain (BRA/PR-378-1335/2020-N) came from a herd sampled in 2020. Regarding the S1 protein gene, the nt sequence was obtained from the same strains and herds sampled in 2018 (BRA/PR-227-840/2018-S1) and in 2019 (BRA/PR-323-425/2019-S1). Unfortunately, it was not possible to obtain sufficient quality for sequencing the S1 gene of the BCoV strain identified in 2020.

### Analysis of the Partial N Gene

The partial N gene nt sequences of the five BCoV strains described herein were compared with 43 enteric and respiratory strains available in GenBank, including South American (Brazilian), North American, European, and Asian strains, which showed nt identities ranging from 94 to 98.5% (Supplementary Table S2). Comparative analysis between the BCoV strains identified herein revealed 98.5 to 100% nt identity with each other. The strains identified intraherd showed 100% identity with each other. Additionally, the BCoV strains described in this study exhibited 97.5 to 98.2% of nt identity with other respiratory Brazilian BCoV strains previously described (GenBank accession numbers MT350497 and MT350498, and MT350500 to MT350505) and 97.5 to 98.2% nt identity with the Mebus, Quebec, and Kakegawa BCoV prototype strains (GenBank accession numbers U00735, AF20295, and AB354579, respectively).

**Figure 1:**
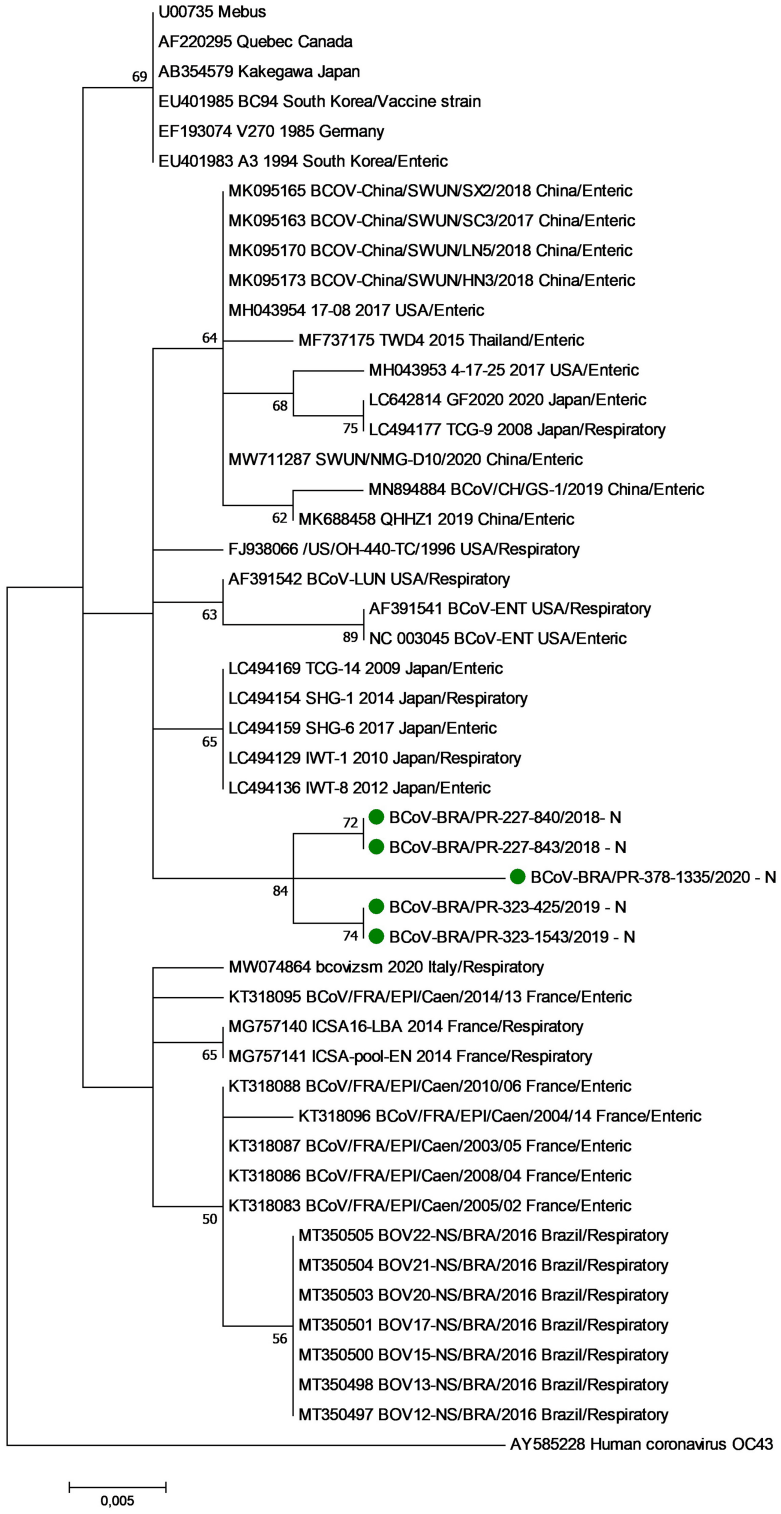
Phylogenetic analysis by the maximum likelihood method of partial (285 nt) N gene of bovine coronavirus strains. The evolutionary history was inferred by using the maximum likelihood method based on the general time reversible model. The percentage of trees in which the associated taxa clustered together is shown next to the branches. The tree is drawn to scale, with branch lengths measured in the number of substitutions per site. The analysis involved 48 nucleotide sequences of bovine coronavirus. A human coronavirus OC43 sequence was used as an outgroup. Evolutionary analyses were conducted in MEGA7. The BCoV strains identified in this study are marked with green filled circles.

The phylogenetic analysis of nt sequences from the partial N gene revealed three major groups (Clusters I, II, and III) (Figure 1). Cluster I consist of historical BCoV strains, including the Mebus, Quebec, and Kakegawa reference strains. The five respiratory BCoV strains identified in this study were grouped in cluster II together with BCoV strains described in Asia and the USA. Finally, cluster III is formed by European BCoV strains and other previously reported Brazilian respiratory BCoV strains.

### Analysis of the Partial S1 Gene

The nt analysis of the partial S1 gene was performed based on the classification into genotypes previously proposed, using a cutoff value of 99.0% nt identity (34). The nt sequences from representative BCoV strains of genotypes #1 to #14 were analyzed, including the reference strains Mebus, Quebec, and Kakegawa. BCoV strains derived from different continents (America, Europe, and Asia) were also analyzed. Additionally, three nt sequences of Brazilian BCoV strains obtained from neonatal calf diarrhea were also included in the analysis (19).

When compared to other 29 BCoV strains available in GenBank the nt and aa sequences of the BRA/PR-227-840 and BRA/PR-323-425 BCoV strains identified in this study showed nt identities ranging from 96.7 to 99% (95.6 to 98.7% for aa deducted sequences) (Supplementary Table S3). Comparative analysis between the strains identified herein revealed 98.9% nt identity with each other (98% for aa sequences). Additionally, these BCoV strains demonstrated 98.8 to 99% nt identity (98.4 to 98.7% for aa deducted sequence) with other Brazilian BCoV strains identified in bovine neonatal diarrhea (GenBank accession numbers DQ479421-DQ479423) previously reported (20) and 97.1 to 98.2% nt (95.9 to 96.9% for aa deducted sequences) identity with the Mebus, Quebec, and Kakegawa BCoV prototype strains (GenBank accession numbers U00735, AF20295, and AB354579, respectively). Nucleotide insertions or deletions were not detected.

**Figure 2:**
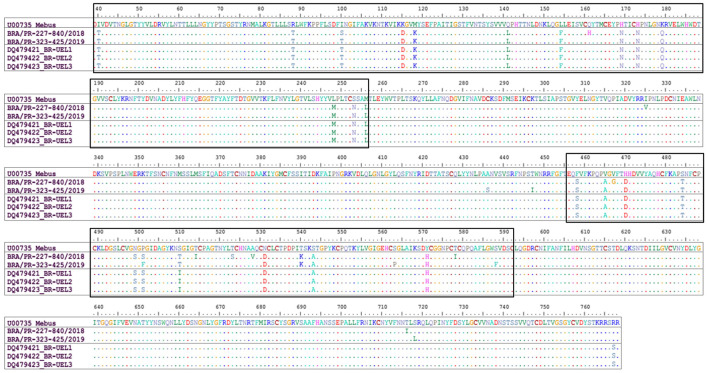
Alignment of the deduced amino acid (39-768 aa concerning the Mebus prototype strain) sequences of the S gene of bovine coronavirus strains. Rectangles show the amino acid changes in two domains observed in the Brazilian strains in this study. The first domain comprises the region of 40-256 deduced amino acid residues. The second domain comprises the hypervariable region between 456-592 deduced amino acid residues.

Comparing the Mebus prototype strain with the two BCoV field strains identified in this study, 23 aa substitutions were identified in both strains. The most of these aa differences were found in two domains, between 40-256 aa, and in the classical hypervariable region, between 456-592 deduced residues. Concerning the first domain (40-256 aa), 13 variations were found in common between the two strains obtained in this study and the Mebus strain and one replacement only entered the BRA/PR-227-840/2018 strain. In the hypervariable region (456-592 aa) 10 aa substitutions were found between the two strains obtained in this study and the Mebus strain, another six specifics substitutions for the BRA/PR-227-840/2018 strain, and two other substitutions for the BRA/PR-323-425/2019 strain. In addition, analyzing the whole fragment (730 aa), another eight and six aa substitutions were observed only in the BRA/PR-227-840/2018 and BRA/PR-323-425/2019 strains, respectively. In the evaluated fragment, 33 cysteine residues were found, however no substitution was observed. When the strains identified in this study were compared with the oldest Brazilian strains, an aa residue substitution was common to both strains, another eight specific substitutions with the BRA/PR-227-840/2018 strain, and six aa substitutions with the BRA/PR-323-425/2019 strain (Figure 2).

**Figure 3:**
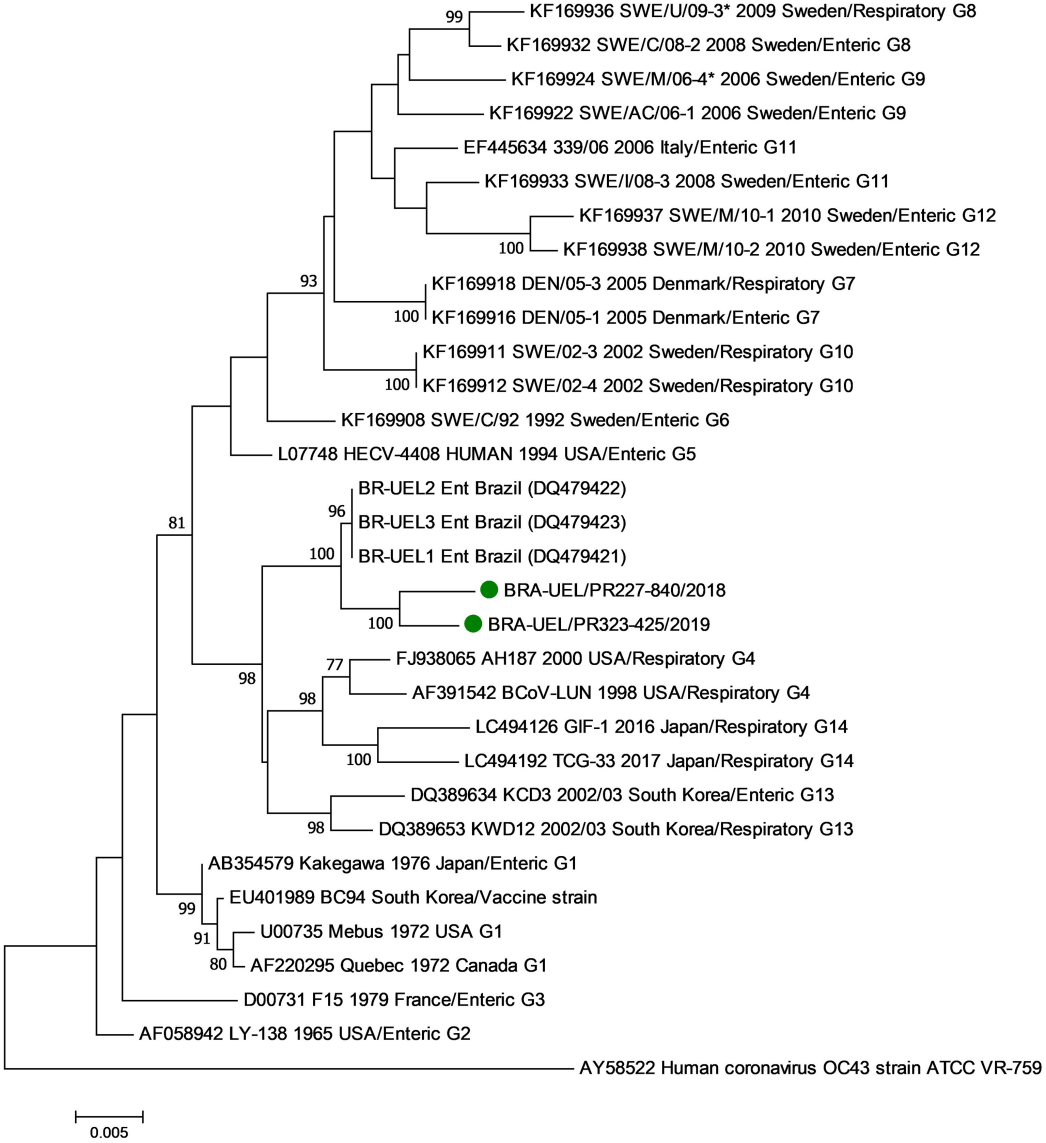
Phylogenetic analysis by the maximum likelihood method of the partial (2581 nt) S gene of bovine coronavirus strains. The evolutionary history was inferred by using the maximum likelihood method based on the general time reversible model. The percentage of trees in which the associated taxa clustered together is shown next to the branches. The tree is drawn to scale, with branch lengths measured in the number of substitutions per site. The analysis involved 29 nucleotide sequences of bovine coronavirus. A human coronavirus OC43 sequence was used as an outgroup. Evolutionary analyses were conducted in MEGA7. The BCoV strains identified in this study are marked with green filled circles.

In the phylogenetic analysis of the S1 gene, the Brazilian strains BRA/PR-227-840 and BRA/PR-323-425 grouped with older Brazilian BCoV sequences forming a new branch (Figure 3). Evaluating the disposition of the oldest Brazilian strains and the dispositions seen in this study in the phylogenetic tree, in addition to the observed identity data of nt (>98.8%) and aa (>98.4%), it can be suggested that the five Brazilian BCoV strains analyzed, belong to a new genotype (#15). In addition, clusters consistent with the type of disease (respiratory/enteric) were not observed.

## Discussion

In this study, high rates of heifer calves and dairy cattle herd BCoV positivity were found. It is known that BCoV is endemic in cattle herds around the world (12). However, few Brazilian surveys report the detection of respiratory BCoV strains in bovines (23, 31, 32). In dairy cattle, only one study describes the association of BCoV and BRD. This recent report, carried out in the same Brazilian state, evaluated bronchoalveolar lavage fluid from calves and detected 40% (6/15) positivity for BCoV in animals with signs of BRD and 16.7% (1/6) in asymptomatic animals. However, none of these infections were singular, all calves had mixed infections with other viruses and/or bacteria, including *P. multocida* (6). Two other studies performed in BRD outbreaks in beef cattle feedlots detected BCoV in the nasopharyngeal swabs of confined cattle during BRD outbreaks (21, 22). Although still very scarce, taken together, these data suggest that BCoV circulates in Brazilian beef and dairy cattle herds and therefore requires attention due to its potential to cause respiratory diseases.

Among bacterial agents, *P. multocida* and *M. haemolytica* colonize the epithelium of the upper respiratory tract, composing the normal microbiota. However, both have a series of virulence factors that favor immunological escape and thus facilitate the development of infection in the lower respiratory tract (35). Some studies also report mixed respiratory infections, such as BCoV and *P. multocida* and/or BCoV and *M. haemolytica* (6, 36, 37). In this report, other forms of association were also identified, such as the triple association of BCoV, *P. multocida*, and *M. haemolytica*, double association of BCoV and *P. multocida*, and with a lower frequency, the double association of BCoV and *M. haemolytica*. The clinical disease caused by these bacteria in cattle results from a combination of stress, immunosuppression, and viral infection that can lead to abundant multiplication in the lower respiratory tract (38).

It is known that BCoV is shed both in respiratory secretions and in feces of naturally or experimentally infected animals. In addition, shedding time is variable and viral RNA can be detected in nasal and/or fecal samples for weeks or months post-infection (39, 40). In the dairy cattle herds evaluated in this study, symptomatic calves were kept in the same pens as the asymptomatic calves and shared automatic feeders. Therefore, the transmission of pathogens can be facilitated between infected animals kept in the same environment due to direct contact between animals and even indirectly by aerosols and contamination of facilities and equipment. In addition, infected animals eliminate the virus intermittently and respiratory reinfections can occur consecutively (25), which may explain the maintenance of BCoV in positive herds.

BCoV is eliminated at a high load in calves with respiratory and enteric disorders and viral shedding can also occur in asymptomatic animals (39, 40). In this study, the presence or absence of clinical signs compatible with BRD at the time of collection does not seem to have interfered with BCoV RNA detection rates since the percentage of positive animals was similar in symptomatic and asymptomatic calves. Based on these results and considering that the sampling was cross-sectional, that is, a single collection was carried out in each herd, three possible explanations can be suggested to justify the high number of asymptomatic BCoV-positive calves. i) Early infection: The infection of animals in the herd may have occurred at different times, and therefore, some animals had not yet shown clinical respiratory signs. ii) Persistent infection: The respiratory tissues of heifer calves already recovered from DRB but still eliminated the virus. iii) Subclinical infection: The infected animals and carriers did not manifest clinical signs of respiratory infection by BCoV. Calves with any of these three forms of infections described above can interfere with the epidemiology of BRD, acting as a source of infection for other susceptible animals in the same herd, especially newborn calves (12).

All dairy cattle herds evaluated had heifer calves with some clinical signs of mild BRD, and 51.2% of the nasal swabs collected were from symptomatic animals. Despite the wide range of microorganisms (viruses and bacteria) involved in the etiology of BRD, the main and frequent potential pathogens detected in respiratory conditions in suckling calves were evaluated in this study using molecular techniques that have high sensitivity. However, even with the clinical diagnosis of BRD in the 10 herds, the only microorganisms identified in the sampling were BCoV, *P. multocida*, and *M. haemolytica*, suggesting a possible association of these pathogens with the clinical respiratory signs observed in the group of symptomatic animals.

In the phylogenetic analyses of both the N and S1 genes, no variations were observed in relation to the origin of the BCoV strains based on respiratory or enteric disease, reinforcing the hypothesis that the same BCoV strain can have dual tropism (21, 41).

The nt sequences of the N gene from the Brazilian BCoV strains identified in this study and seven strains from another study (unpublished) were arranged in two different clusters. Interestingly, the Brazilian BCoV strains in both studies come from the same Brazilian state, but from different regions that are approximately 600 km apart. These findings reinforce that the genetic variability of the strains is related to detection in different years and geographic regions, and therefore suggest that there are different strains of BCoV circulating in Brazil, even in the same state. Additionally, these findings revealed the genetic diversity even from an internal protein in the viral particle.

In the analysis of the S1 gene, it was observed that contemporary and older Brazilian BCoV strains are genetically distant from strains described in other countries and included in the genotype classification analysis (>2.2% and 2.1% at the nt and aa levels, respectively), therefore suggesting that our strains belong to another genotype (called #15). Based on the analyses described above and added to previously published data, it is not surprising that BCoV strains from different regions or countries are grouped into different clusters (except for three genotypes #1, #4, and #11) (34). However, even considering the same genotype, the analysis of contemporary and older Brazilian strains revealed subgroups in different clusters according to the geographical and temporal distribution of the strains. In summary, these findings reinforce the need for molecular studies to better understand the epidemiology of coronavirus infections and for the implementation of assertive prophylactic measures aimed at reducing BCoV infection disorders.

An essential tool for the prevention of respiratory disorders is the use of vaccines against the main viral and bacterial pathogens causing respiratory diseases, including BCoV. However, to date, commercial vaccines against BRD pathogens containing BCoV-derived antigens are not available in Brazil. Currently, only vaccines containing BCoV in combination with other enteric agents are available to prevent neonatal diarrhea in calves (23). However, in the hypervariable domain of the S1 gene analysis showed high differences between the Brazilian BCoV field strains and the Mebus reference of strain. Given the antigenic differences observed between the strains currently detected worldwide, including the strains in this study, and the reference vaccine Mebus strain, it is possible that the vaccines may have reduced effectiveness against strains that are currently circulating in Brazilian herds (21, 42, 43). Nevertheless, more studies focused on molecular epidemiology are important to understand the genetic variability of respiratory and enteric BCoV and thus assess the implications of antigenic diversity in relation to viral pathogenicity and immunoprophylactic aspects.

Finally, this study describes of diagnosis of BCoV, *P. multocida*, and *M. haemolytica* in the upper respiratory tract of heifer calves from high-production dairy cattle herds with and without clinical signs of respiratory disease. Additionally, the molecular characterization of the BCoV strains identified in this study shows that they are ancestrally different from the prototype strains, strains previously reported in Brazil, and even from strains reports in other part of the studied region. Continuous studies focusing on multietiological diagnosis and molecular characterization are needed to provide an understanding of infections and the implementation of assertive prophylactic measures aimed at reducing respiratory disorders in calves. Together, these actions can contribute to reducing the frequency and intensity of BRD in dairy cattle herds and increasing the productive indices of the Brazilian milk production chain.

## Data Availability Statement

GenBank accession numbers of strains of bovine coronavirus N gene: BRA/PR-227-840/2018-N, BRA/PR-227-843/2018-N, BRA/PR-323-425/2019-N, BRA/PR-323-1543/2019-N, and BRA/PR-378-1335/2020-N (OM632709- OM632713); bovine coronavirus S1 gene: BRA/PR-227-840/2018 and BRA/PR-323-425/2019 (OM632714 and OM632715); Pasteurella multocida BRA/PR-323-1543/2019 (OM632716); and Mannheimia haemolytica BRA/PR-378-42/43/2020 (OM632717).

## Ethics Statement

This study was approved by the Ethics Committee on Animal Use (CEUA) of Universidade Estadual de Londrina (CEUA protocol n° 1835.2019.45). Written informed consent was obtained from the owners for the participation of their animals in this study.

## Author Contributions

AAA: design and project administration. APSF, DEB, and EAB: methodology. APSF and AMD: data curation, formal analysis, and writing—original draft preparation. AAA and AFA: manuscript editing and revision. All authors have read and agreed to the published version of the manuscript.

## Funding

This work was supported by the National Institute of Science and Technology of Dairy Production Chain (CNPq/INCT-Leite) [grant number 465725/2014-7].

## Conflict of Interest

The authors declare that the research was conducted in the absence of any commercial or financial relationships that could be construed as a potential conflict of interest.

## Publisher's Note

All claims expressed in this article are solely those of the authors and do not necessarily represent those of their affiliated organizations, or those of the publisher, the editors and the reviewers. Any product that may be evaluated in this article, or claim that may be made by its manufacturer, is not guaranteed or endorsed by the publisher.
