# Supplementary material for: Bovine Coronavirus Co-infection and Molecular Characterization in Dairy Calves With or Without Clinical Respiratory Disease
Source: Front Vet Sci. 2022 May 25;9:895492. doi: 10.3389/fvets.2022.895492 (PMC9174899; doi:10.3389/fvets.2022.895492)
Supplement: Supplementary file 1 [file Data_Sheet_1.docx]

Bovine coronavirus co-infection and molecular characterization in dairy calves with or without clinical respiratory disease

Ana Paula S. Frucchi, Alais M. Dall Agnol, Dalton E. Bronkhorst, Edsel A. Beuttemmuller, Amauri A. Alfieri, Alice F. Alfieri

Supplementary Material

| **Herd** | **Year** | **N°**  **calves** | **Virus^1^ (%)** | | |  | **Bacteria^2^ (%)** | | | | | | |
| --- | --- | --- | --- | --- | --- | --- | --- | --- | --- | --- | --- | --- | --- |
|  |  |  | **BCoV positive** | | |  | ***P. multocida*** | | |  | ***M. haemolytica*** | | |
|  |  |  | **BRD calves** | **Control calves** | **Total** |  | **BRD calves** | **Control calves** | **Total** |  | **BRD calves** | **Control calves** | **Total** |
| A | 2018 | 35 | 16/18 | 16/17 | 32 (91.4) |  | 17/18 | 13/17 | 30 (85.7) |  | 14/18 | 13/17 | 27 (77.1) |
| B | 2018 | 16 | 3/8 | 5/8 | 8 (50.0) |  | 1/8 | 0/8 | 1 (6.3) |  | 0/8 | 0/8 | ̶ |
| C | 2018 | 10 | 0/6 | 1/4 | 1 (10.0) |  | 0/6 | 0/4 | ̶ |  | 0/6 | 0/4 | ̶ |
| D | 2018 | 9 | 4/7 | 0/2 | 4 (44.4) |  | 3/7 | 0/2 | 3 (33.3) |  | 0/7 | 0/2 | ̶ |
| E | 2018 | 6 | 0/5 | 0/1 | ̶ |  | 0/5 | 1/1 | 1 (16.7) |  | 3/5 | 0/1 | 3 (50.0) |
| F | 2018 | 7 | 5/5 | 0/2 | 5 (71.4) |  | 1/5 | 0/2 | 1 (14.3) |  | 0/5 | 0/2 | ̶ |
| G | 2019 | 13 | 1/7 | 0/6 | 1 (7.7) |  | 1/7 | 0/6 | 1 (7.7) |  | 0/7 | 0/6 | ̶ |
| H | 2019 | 6 | 3/4 | 2/2 | 5 (83.3) |  | 3/4 | 2/2 | 5 (83.3) |  | 0/4 | 0/2 | ̶ |
| J | 2020 | 37 | 9/13 | 17/24 | 26 (70.3) |  | 9/13 | 14/24 | 23 (62.2) |  | 7/13 | 18/24 | 25 (67.6) |
| K | 2020 | 27 | 5/12 | 6/15 | 11 (40.7) |  | 0/12 | 1/15 | 1 (3.7) |  | 0/12 | 0/15 | ̶ |
| **Total** | | **166** | **46/85 (54.1)** | **47/81 (58.0)** | **93 (56.0)** |  | **35/85 (41.2)** | **31/81 (38.3)** | **61 (39.8)** |  | **24/85 (28.2)** | **31/81 (38.3)** | **55 (33.1)** |

**Table S1**. Infectious agents identified by molecular assays in the upper respiratory tract of heifer calves, with and without (controls) bovine respiratory disease (BRD) clinal signs, from high production dairy cattle herds.

**^(1)^** BCoV (bovine coronavirus). All evaluated samples were negative for: bovine alphaherpesvirus 1; bovine viral diarrhea virus; bovine parainfluenza virus 3; and bovine respiratory syncytial virus.

**^(2)^** *P. multocida* (*Pasteurella multocida*) and *M. haemolytica* (*Mannheimia haemolytica*). All evaluated samples were negative for *Histophilus somni* and *Mycoplasma bovis*

| **GenBank accession number – Sequence name** | **BCoV Brazilian strains – Percentages of nt identity** | | | | |
| --- | --- | --- | --- | --- | --- |
|  | BRA/PR-227-840/2018 | BRA/PR-227-843/2018 | BRA/PR-323-425/2019 | BRA/PR-323-1543/2019 | BRA/PR-378-1335/2020 |
| BRA/PR-227-840/2018 | ID | 100 | 99.2 | 99.2 | 98.5 |
| BRA/PR-227-843/2018 | 100 | ID | 99.2 | 99.2 | 98.5 |
| BRA/PR-323-425/2019 | 99.2 | 99.2 | ID | 100 | 98.5 |
| BRA/PR-323-1543/2019 | 99.2 | 99.2 | 100 | ID | 98.5 |
| BRA/PR-378-1335/2020 | 98.5 | 98.5 | 98.5 | 98.5 | ID |
| MT350505_BOV22-NS/BRA/2016 | 98.2 | 98.2 | 98.2 | 98.2 | 97.5 |
| MT350504_BOV21-NS/BRA/2016 | 98.2 | 98.2 | 98.2 | 98.2 | 97.5 |
| MT350503_BOV20-NS/BRA/2016 | 98.2 | 98.2 | 98.2 | 98.2 | 97.5 |
| MT350501_BOV17-NS/BRA/2016 | 98.2 | 98.2 | 98.2 | 98.2 | 97.5 |
| MT350500_BOV15-NS/BRA/2016 | 98.2 | 98.2 | 98.2 | 98.2 | 97.5 |
| MT350498_BOV13-NS/BRA/2016 | 98.2 | 98.2 | 98.2 | 98.2 | 97.5 |
| MT350497_BOV12-NS/BRA/2016 | 98.2 | 98.2 | 98.2 | 98.2 | 97.5 |
| U00735_Mebus | 98.2 | 98.2 | 98.2 | 98.2 | 97.5 |
| AF220295_Quebec | 98.2 | 98.2 | 98.2 | 98.2 | 97.5 |
| AB354579_Kakegawa | 98.2 | 98.2 | 98.2 | 98.2 | 97.5 |
| EU401985_BC94 | 98.2 | 98.2 | 98.2 | 98.2 | 97.5 |
| AF391542_BCoV-LUN | 98.5 | 98.5 | 98.5 | 98.5 | 97.8 |
| AF391541_ BCoV-ENT | 97.8 | 97.8 | 97.8 | 97.8 | 97.1 |
| FJ938066_/US/OH-440-TC/1996 | 98.5 | 98.5 | 98.5 | 98.5 | 97.8 |
| MH043954_17-08 | 98.5 | 98.5 | 98.5 | 98.5 | 97.8 |
| MH043953_4-17-25 | 97.8 | 97.8 | 97.8 | 97.8 | 97.1 |
| NC_003045_BCoV-ENT | 97.8 | 97.8 | 97.8 | 97.8 | 97.1 |
| MW074864_bcovizsm | 97.8 | 97.8 | 97.8 | 97.8 | 97.1 |
| KT318083_BCoV/FRA/EPI/Caen/2005/02 | 94.7 | 94.7 | 94.7 | 94.7 | 94.0 |
| KT318086_BCoV/FRA/EPI/Caen/2008/04 | 97.8 | 97.8 | 97.8 | 97.8 | 97.1 |
| KT318087_BCoV/FRA/EPI/Caen/2003/05 | 97.8 | 97.8 | 97.8 | 97.8 | 97.1 |
| KT318088_BCoV/FRA/EPI/Caen/2010/06 | 97.8 | 97.8 | 97.8 | 97.8 | 97.1 |
| KT318095_BCoV/FRA/EPI/Caen/2014/13 | 97.8 | 97.8 | 97.8 | 97.8 | 97.1 |
| KT318096_BCoV/FRA/EPI/Caen/2004/14 | 97.5 | 97.5 | 97.5 | 97.5 | 96.8 |
| MG757140_ICSA16-LBA | 98.5 | 98.5 | 98.5 | 98.5 | 97.8 |
| MG757141_ICSA-pool-EN_ | 98.5 | 98.5 | 98.5 | 98.5 | 97.8 |
| EF193074_V270 | 98.2 | 98.2 | 98.2 | 98.2 | 97.5 |
| MK095173_BCOV-China/SWUN/HN3/2018 | 98.5 | 98.5 | 98.5 | 98.5 | 97.8 |
| MK095170_BCOV-China/SWUN/LN5/2018 | 98.5 | 98.5 | 98.5 | 98.5 | 97.8 |
| MK095165_BCOV-China/SWUN/SX2/2018 | 98.5 | 98.5 | 98.5 | 98.5 | 97.8 |
| MK095163_BCOV-China/SWUN/SC3/2017 | 98.5 | 98.5 | 98.5 | 98.5 | 97.8 |
| EU401983_A3 | 98.2 | 98.2 | 98.2 | 98.2 | 97.5 |
| MF737175_TWD4_2015 | 98.2 | 98.2 | 98.2 | 98.2 | 97.5 |
| MN894884_BCoV/CH/GS-1/2019 | 97.8 | 97.8 | 97.8 | 97.8 | 97.1 |
| MK688458_QHHZ1_2019 | 98.2 | 98.2 | 98.2 | 98.2 | 97.5 |
| MW711287_SWUN/NMG-D10/2020 | 98.5 | 98.5 | 98.5 | 98.5 | 97.8 |
| LC642814_GF2020 | 97.8 | 97.8 | 97.8 | 97.8 | 97.1 |
| LC494177_TCG-9 | 97.8 | 97.8 | 97.8 | 97.8 | 97.1 |
| LC494169_TCG-14 | 95.4 | 95.4 | 95.4 | 95.4 | 94.7 |
| LC494154_SHG-1 | 95.4 | 95.4 | 95.4 | 95.4 | 94.7 |
| LC494159_SHG-6 | 98.2 | 98.2 | 98.2 | 98.2 | 97.5 |
| LC494129_IWT-1 | 98.2 | 98.2 | 98.2 | 98.2 | 97.5 |
| LC494136_IWT-8 | 95.4 | 95.4 | 95.4 | 95.4 | 94.7 |

**Table S2**. Percentages of identity to the partial (285 nt) nucleotide sequences of the N gene of bovine coronavirus field strains identified in this study compared to 43 bovine coronavirus strains.

**Table S3**. Percentages of identity to the partial nucleotide (2581 nt) and amino acid (855 aa) sequences of the S1 gene of bovine coronavirus field strains identified in this study compared to 29 bovine coronavirus strains.

| **GenBank accession number – Sequence name** | **Genotype** | **Year** | **Country** | **BCoV Brazilian strains**  **Percentages of nt identity (aa)** | |
| --- | --- | --- | --- | --- | --- |
|  |  |  |  | **BRA-UEL/PR227-840/2018** | **BRA-UEL/PR323-425/2019** |
| BRA-UEL/PR227-840/2018 | 15 | 2018 | Brazil | ID | 98.9 (98) |
| BRA-UEL/PR323-425/2019 | 15 | 2019 | Brazil | 98.9 (98) | ID |
| DQ479421_BR-UEL1 | 15 | 2006 | Brazil | 98.8 (98.4) | 99.0 (98.7) |
| DQ479422_BR-UEL2 | 15 | 2006 | Brazil | 98.8 (98.4) | 99.0 (98.7) |
| DQ479423_BR-UEL3 | 15 | 2006 | Brazil | 98.8 (98.4) | 99.0 (98.7) |
| U00735_Mebus | 1 | 1972 | USA | 97.1 (95.9) | 97.3 (96.3) |
| AF220295_Quebec | 1 | 1972 | Canada | 97.1 (95.9) | 97.3 (96.3) |
| AB354579_Kakegawa | 1 | 1976 | Japan | 97.4 (96.4) | 97.6 (96.9) |
| EU401989_BC94 | 1 | - | South Korea | 97.2 (96.2) | 97.4 (96.6) |
| AF058942_LY-138 | 2 | 1965 | USA | 97.3 (96.7) | 97.3 (96.8) |
| D00731_F15_1979 | 3 | 1979 | France | 96.9 (96.1) | 97.1 (96.8) |
| FJ938065_AH187 | 4 | 2000 | USA | 97.8 (97.4) | 97.9 (97.8) |
| AF391542_BCoV-LUN | 4 | 1998 | USA | 97.7 (97.5) | 97.9 (98.1) |
| L07748_HECV-4408_HUMAN | 5 | 1994 | USA | 97.5 (96.4) | 97.7 (97.0) |
| KF169908_SWE/C/92 | 6 | 1992 | Sweden | 97.3 (96.8) | 97.5 (97.3) |
| KF169918_DEN/05-3 | 7 | 2005 | Denmark | 97.1 (96.4) | 97.3 (97.1) |
| KF169916_DEN/05-1 | 7 | 2005 | Denmark | 97.1 (96.4) | 97.3 (97.1) |
| KF169936_SWE/U/09-3* | 8 | 2009 | Sweden | 97.1 (96.7) | 97.1 (96.7) |
| KF169932_SWE/C/08-2 | 8 | 2008 | Sweden | 97.1 (96.4) | 97.3 (97.1) |
| KF169924_SWE/M/06-4* | 9 | 2006 | Sweden | 97.1 (96.2) | 97.1 (96.4) |
| KF169922_SWE/AC/06-1 | 9 | 2006 | Sweden | 97.2 (96.7) | 97.3 (96.8) |
| KF169911_SWE/02-3 | 10 | 2002 | Sweden | 97.4 (97.4) | 97.5 (97.8) |
| KF169912_SWE/02-4 | 10 | 2002 | Sweden | 97.4 (97.4) | 97.5 (97.8) |
| KF169933_SWE/I/08-3 | 11 | 2008 | Sweden | 97.4 (96.8) | 97.3 (96.9) |
| EF445634_339/06_2006 | 11 | 2006 | Italy | 97.2 (97) | 97.3 (97.4) |
| KF169937 SWE/M/10-1 | 12 | 2010 | Sweden | 96.7 (95.6) | 96.8 (96.1) |
| KF169938_SWE/M/10-2 | 12 | 2010 | Sweden | 96.7 (96.0) | 96.8 (96.2) |
| DQ389634_KCD3_2002/03 | 13 | 2002 | South Korea | 97.7 (97.5) | 97.7 (97.6) |
| DQ389653 KWD12_2002/03 | 13 | 2002 | South Korea | 97.8 (97.5) | 97.7 (97.6) |
| LC494126_GIF-1 | 14 | 2016 | Japan | 97.4 (97.7) | 97.4 (98.1) |
| LC494192_TCG-33 | 14 | 2017 | Japan | 97.4 (98.0) | 97.3 (98.1) |
